# Experimental Research on Crack Resistance of Steel–Polyvinyl Alcohol Hybrid Fiber-Reinforced Concrete

**DOI:** 10.3390/ma17133097

**Published:** 2024-06-25

**Authors:** Jingjiang Wu, Wenjie Zhang, Juhong Han, Zheyuan Liu, Jie Liu, Yafei Huang

**Affiliations:** 1 China Construction Seventh Engineering Division Co., Ltd., Zhengzhou 450003, China; wujingjiang@cscec.com (J.W.); liujie@cscec.com (J.L.); huangyafei@cscec.com (Y.H.); 2 CCCC Second Harbour Engineering Co., Ltd., Wuhan 430040, China; 3 School of Water Conservancy and Transportation, Zhengzhou University, Zhengzhou 450001, China; 13015112337@163.com

**Keywords:** steel fiber, PVA fiber, crack resistance, analytic hierarchy process

## Abstract

This paper investigates the effects of steel fiber and PVA fiber hybrid blending on the compressive strength ($f_{cc}$), splitting tensile strength ($f_{ts}$), compression energy ($W_{1.0}$), and shrinkage properties of concrete. It also establishes a multi-factor crack resistance index evaluation model based on the Analytic Hierarchy Process (AHP) to comprehensively evaluate the crack resistance of concrete. The results show that the steel–PVA hybrid fiber (S-PVA HF) further enhances $f_{cc}$, $f_{ts}$, the compression energy, and the shrinkage suppression properties of the concrete. The crack resistance of the steel–PVA hybrid fiber concrete (S-PVA HFRC) is the best when the proportion of steel fiber is 1.0% and that of the PVA fiber is 0.2%, and it increases up to 143% compared to the baseline concrete. The established concrete crack resistance evaluation model has a certain reliability.

## 1. Introduction

A concrete-faced rockfill dam (CFRD) is a structure where the rockfill body is the primary support structure and the concrete panel serves as the primary anti-seepage structure [1]. However, the thin-wall structure of the panel tends to generate cracks, and excessive deformation of the dam body leads to structural cracks and compressive failure of the panel [2]. In the early stage of concrete pouring, the tensile strength of the panel concrete is low, and cracks are caused when the tensile stress generated by $\varepsilon_{t}$ and $\varepsilon_{d}$ exceeds its own tensile strength limit [1,3,4]. Cracks in the concrete panel can damage the structural integrity of the concrete panel, negatively affecting the durability of the concrete panel and the normal operation of the CFRD [5]. In response to the explicit requirements put forward by the “*Fourteenth Five-Year Plan for Water Safety Guarantee*” [6], reducing the cracks in the concrete panel and maintaining the stability of the overall structure are important issues in the design and construction of a CFRD. Considering the compressive failure of the concrete panel and cracking due to concrete shrinkage, improving the crack resistance of concrete is a crucial matter.

Improving the crack resistance of concrete primarily involves enhancing its compressive performance, tensile performance, and the ability to inhibit $\varepsilon_{d}$ and $\varepsilon_{t}$, among others. The most commonly used and effective method is the incorporation of fibers [7,8,9], which include common types such as steel fiber and polyvinyl alcohol (PVA) fiber and other polymer fibers [10,11].

Extensive research has been conducted on the impact of steel fiber and PVA fiber on concrete performance. Steel fiber can not only enhance the mechanical properties of concrete but also reduce concrete shrinkage and improve crack resistance. Many scholars have conducted studies on the enhancement of concrete compression, tensile, and axial compressive mechanical properties by steel fibers [12,13,14,15,16,17,18,19]. It has been concluded that steel fibers can cross cracks, restrict their further development, thereby enhancing the mechanical properties of concrete. Some scholars have concluded that the shrinkage rate of concrete decreases with the addition of steel fibers. B. Bandelj [20] found that the $\varepsilon_{d}$ and $\varepsilon_{t}$ of concrete were minimal with a 1.0% addition of 30 mm long end-hooked steel fibers. B. Miao [21] found that the $\varepsilon_{d}$ and $\varepsilon_{t}$ of concrete showed a linear decreasing trend as the quantity of steel fiber increased from 0% to 1.5%. Xiaoxia Zheng’s research [22] found that the $\varepsilon_{d}$ and $\varepsilon_{t}$ of concrete decreased with the addition of shear-type steel fibers and wavy steel fibers. Shen [23] researched the influence of 60 mm long end-hooked steel fibers (0.3%, 0.6%, 0.9%) on the shrinkage of 30 mm aggregate concrete, and found that compared to ordinary concrete, $\varepsilon_{t}$ decreased by 17.6%, 29.4%, and 64.7%, respectively, with the increase in steel fiber dosage. Shen [24] researched the impact of 60 mm long end-hooked steel fibers (0.12%, 0.24%, and 0.36%) on the shrinkage of 25 mm aggregate concrete, and the results showed that as the quantity of steel fibers increased, concrete shrinkage decreased by 34.9%, 53.9%, and 70.7% respectively. Overall, the incorporation of steel fibers improves the compressive and tensile properties of concrete and reduces concrete shrinkage. However, its effect on restricting microcracks is not ideal, and most research focuses on concrete with an aggregate particle size not exceeding 30 mm.

PVA fiber has high tensile strength, high $E_{f}$, good dispersion within the cement concrete matrix, higher physical anchoring force with cement-based materials, and a large number per unit weight, which can effectively reduce the generation of microcracks and improve the crack resistance of concrete. The incorporation of PVA fiber can improve the compressive and $f_{ts}$ of concrete [25,26,27], reduce concrete shrinkage, and enhance the crack resistance of concrete [28,29,30,31,32,33]. Lei Wang’s research [34] on the impact of PVA fiber, polypropylene (PP) fiber, and polyacrylonitrile (PAN) fiber on panel concrete shrinkage and crack resistance shows that PVA fiber achieves the greatest enhancement in panel concrete crack resistance and best shrinkage reduction effect. In general, PVA fiber improves the crack resistance of panel concrete quite effectively. However, when cracks continue to develop, the control of cracks by PVA fiber will fail.

In summary, both steel fiber and PVA fiber can effectively enhance the crack resistance of concrete, but their improvement mechanisms are different. Steel fiber crossing the cracks prevents the rapid development of cracks. PVA fiber, using the characteristics of its high strength, high $E_{f}$, and large number of polymer fibers, inhibits the generation and development of microcracks. The possibility of mixing steel fiber and PVA fiber to synergistically enhance the crack resistance of panel concrete is worth studying.

At present, most research on S-PVA HFRC is focused on $f_{cc}$, $f_{ts}$, and $f_{c}$ [35,36], with less study in the aspect of concrete crack resistance.

Therefore, this paper combines the different characteristics of steel fibers and PVA fibers for enhancing the crack resistance of concrete. Steel fibers and PVA fibers are mixed together to cooperatively improve the crack resistance of concrete. The testing of crack resistance mainly involves an $f_{cc}$ test, $f_{ts}$ test, uniaxial compression test, and $\varepsilon_{d}$ and $\varepsilon_{t}$ tests. Then, based on the AHP, a crack resistance evaluation model is established to comprehensively evaluate the crack resistance of concrete, thereby determining the optimal mixing ratio of steel fibers and PVA fibers in S-PVA HFRC.

## 2. Experimental Materials and Methods

### 2.1. Experimental Materials

**Figure 1:**
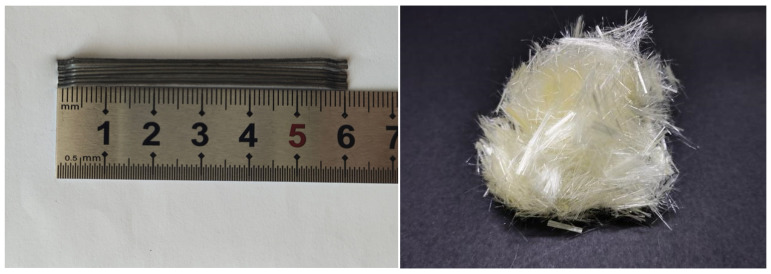
Steel fiber and PVA fiber diagram.

In this experiment, the following raw materials were used, each with its respective parameters and properties: P.O42.5 cement served as the cement type. The coarse aggregate comprised crushed stones ranging from 5 mm to 40 mm, with small stones (5 mm–20 mm) accounting for 55% and large stones (20 mm–40 mm) accounting for 45%. The fine aggregate (the fineness modulus was 2.63) came from natural river sand and continuous grading. Three-dimensional hooked-end steel fibers with a length of 60 mm were used as the steel fibers, as depicted in Figure 1. The PVA fibers utilized were supplied by Kuraray Co., Ltd. (Tokyo, Japan), with a diameter of 31 μm and a length of 12 mm, as shown in Table 1 and Figure 1. A high-efficiency polycarboxylate-based water-reducing agent was employed. High-quality Class I fly ash was utilized. The experiment employed tap water sourced from the local supply.

According to China standard SL228-2013 [37], panel concrete should have a 28-day strength grade of at least C25, a water–binder ratio below 0.45, and fly ash substituting cement should be limited to 30%. In this experiment, China standard DL/T5330-2015 [38] and China standard JG/T 472-2015 [39] were referenced. Nine concrete batches were determined using calculated mix proportions (Table 2).

### 2.2. Experimental Methods

#### 2.2.1. $f_{cc}$ Test and $f_{ts}$ Test

**Figure 2:**
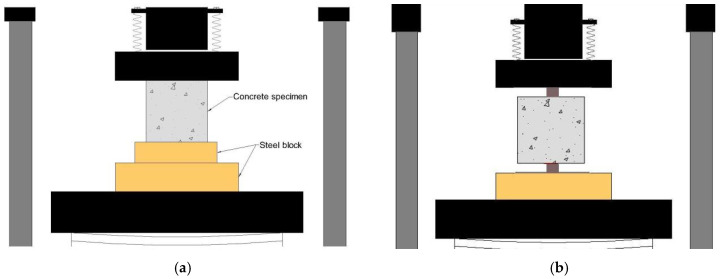
Schematic diagram of concrete $f_{cc}$ test and $f_{ts}$ test. (**a**) Schematic diagram of concrete $f_{cc}$ test. (**b**) Schematic diagram of concrete $f_{ts}$ test.

The $f_{cc}$ test and $f_{ts}$ test of concrete were carried out in accordance with the “*Specification for Hydraulic Concrete Test*” (SL/T352-2020) [40]. Cube specimens with dimensions of 150 mm × 150 mm × 150 mm were demolded after 24 h and then cured for 28 days in a standard curing chamber (RH > 95%, temperature = 20 ± 2 °C). $f_{cc}$ and $f_{ts}$ were measured after curing for 3, 7, 14, and 28 days. Three specimens in each group were prepared under different conditions at different ages, as shown in Figure 2.

#### 2.2.2. Uniaxial Compression Test

**Figure 3:**
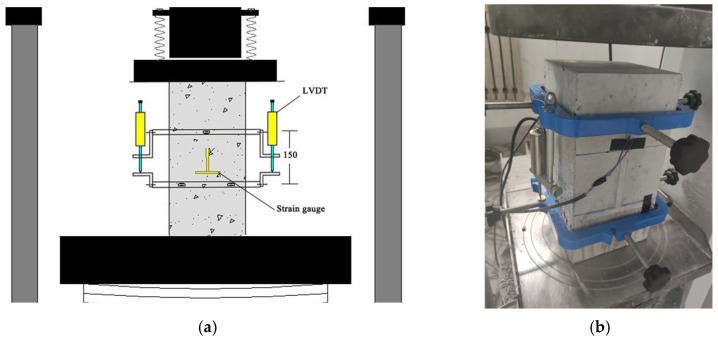
Uniaxial compression test of concrete. (**a**) Schematic diagram of concrete uniaxial compression test. (**b**) Experimental photo of concrete uniaxial compression test.

The uniaxial compression test of concrete was carried out according to the “*Water Conservancy Concrete Test Specification*” (SL/T 352-2020) [40]. The dimensions of the concrete specimen were 150 mm × 150 mm × 300 mm (Figure 3). Six specimens per group were prepared at different ages and conditions, three of which were used to determine the axial compressive strength and the preloading criteria for the full curve test, and the other three were used to determine the full stress–strain curve. The specimen with a size of 150 mm × 150 mm × 300 mm was demolded after 24 h and cured for 28 days in a standard curing chamber (RH > 95%, temperature is 20 ± 2 °C). After 3, 7, 14, and 28 days of curing, the stress–strain curve of the concrete was measured, as shown in Figure 3. According to the test data, $E_{f}$ and $W_{1.0}$ of concrete were calculated, $E_{f}$ was taken as the modulus of elasticity of the cut line with stress from 0.5 MPa to 40% of the destructive stress, calculated according to Formula (1). $W_{1.0}$ was the area under the load–displacement curve for an axial deformation from 0 to L0 × 1.0% mm (L0 is 150 mm).

(1) $$E_{f}=\frac{∆\sigma }{∆\varepsilon }÷1000$$

In the formula: $E_{f}$—the elastic modulus of concrete, ∆σ—stress increase from 0.5 MPa to 40% destructive stress (MPa), ∆ε—increase in strain from 0.5 MPa to 40% of destructive stress.

#### 2.2.3. Autogenous Shrinkage and Drying Shrinkage Test

**Figure 4:**
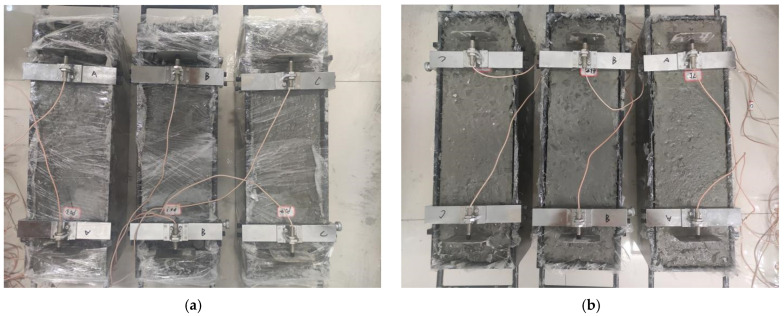
Test diagram of concrete autogenous shrinkage and drying shrinkage. (**a**) Picture of concrete autogenous shrinkage test. (**b**) Picture of concrete drying shrinkage test.

**Figure 5:**
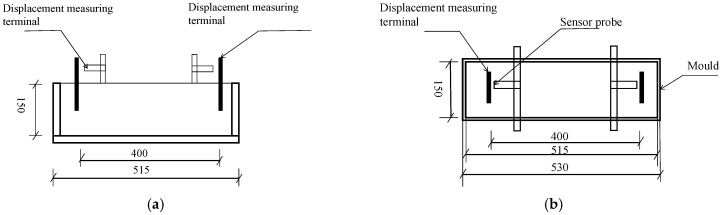
Dimensional drawing of the shrinkage test apparatus. (**a**) Schematic diagram of concrete self-shrinkage test. (**b**) Schematic diagram of concrete drying shrinkage test.

The shrinkage test and measurement of concrete were carried out using a non-contact concrete shrinkage deformation tester, referring to the “*Standard Test Methods for Long-Term Performance and Durability of Ordinary Concrete*” (GB/T 50082-2009) [41], as shown in Figure 4. Three specimens in each group were prepared under different conditions at different ages. The well-cast specimens were left in the laboratory for 4 h, and then cured in a drying room (20 ± 2 °C and 50 ± 5% RH). The specimen size was 150 mm × 150 mm × 515 mm, and the mold size is shown in Figure 5. The $\varepsilon_{t}$ (3 days) and $\varepsilon_{d}$ (28 days) of the concrete were measured.

## 3. Results and Discussion

### 3.1. $f_{cc}$ of Concrete

**Figure 6:**
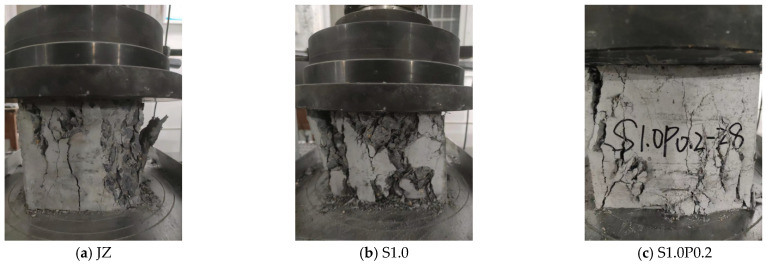
Typical test pictures of concrete $f_{cc}$ at 28 days.

The typical test figure after the $f_{cc}$ test of concrete is shown in Figure 6. Figure 6 shows the failure modes of the reference concrete, concrete with 1.0% steel fiber content, and concrete with 1.0% steel fiber and 0.2% PVA fiber content, respectively.

From Figure 6, we can conclude:

(1) After the compressive failure of the reference concrete, the internal structure of the concrete was severely damaged, the surface was severely peeled off, and the integrity of the matrix was poor.
(2) After the addition of steel fibers, the peeling of concrete was reduced, the integrity of the matrix was improved, but the internal truss system of the concrete could be clearly seen. In the areas the steel fiber truss system did not cover, the concrete still had damage.
(3) The matrix integrity of the S-PVA HFRC was the best. After the PVA fiber was added to SFRC, the matrix integrity was greatly improved. Although the structure of the concrete matrix was damaged, there was little surface peeling.

#### 3.1.1. Variation Trend of $f_{cc}$ in SFRC

**Figure 7:**
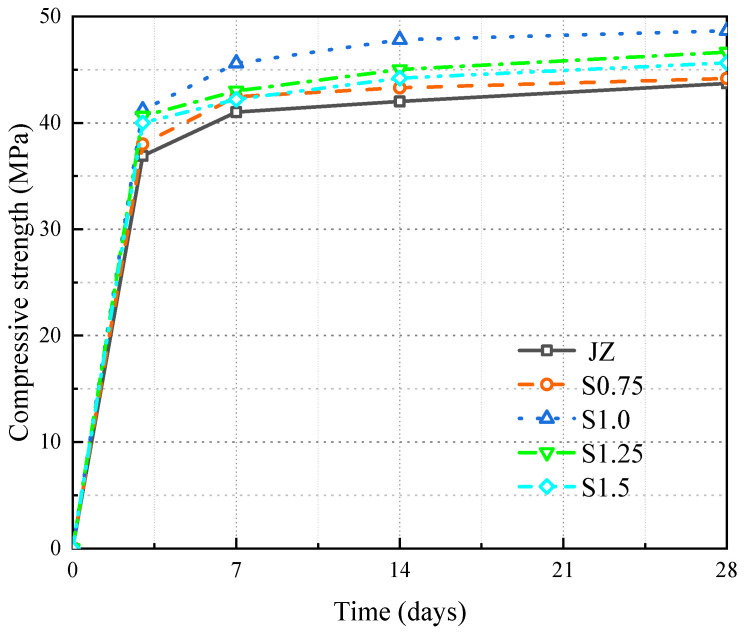
Variation of $f_{cc}$ in SFRC.

The trend of $f_{cc}$ over time for JZ, S0.75, S1.0, S1.25, and S1.5 is shown in Figure 7.

As can be seen from Figure 7:

(1) As the age increased, the $f_{cc}$ of the concrete gradually increased. It was also found that the strength of concrete increased fastest between 3 and 7 days of age. At 7 days, it reached over 80% of the $f_{cc}$ at 28 days.
(2) At each age, as the content of steel fiber increased, the $f_{cc}$ first rose and then fell, peaking when the steel fiber content was 1.0%. At 28 days, compared to the $f_{cc}$ of JZ, S1.0 increased from 43.7 MPa to 48.66 MPa, an increase of 11.4%.

The results show that steel fibers can enhance the $f_{cc}$ of concrete [12,13,14,15,16]. After the concrete is compressed to produce microcracks, the bridging effect of the steel fibers restricts the crack expansion caused by the tensile stress due to expansion, greatly improving the compressive toughness of the specimen. However, when the steel fiber content is greater than 1.0%, the quantity of fibers increases, and the specific surface area that the cement paste needs to wrap increases, leading to more internal defects and a decrease in matrix density in the concrete, and thus the $f_{cc}$ of the concrete also decreases. In summary, for the panel concrete studied in this paper, 1.0% content of steel fibers provides the greatest enhancement in $f_{cc}$.

#### 3.1.2. Variation of $f_{cc}$ in S-PVA HFRC

**Figure 8:**
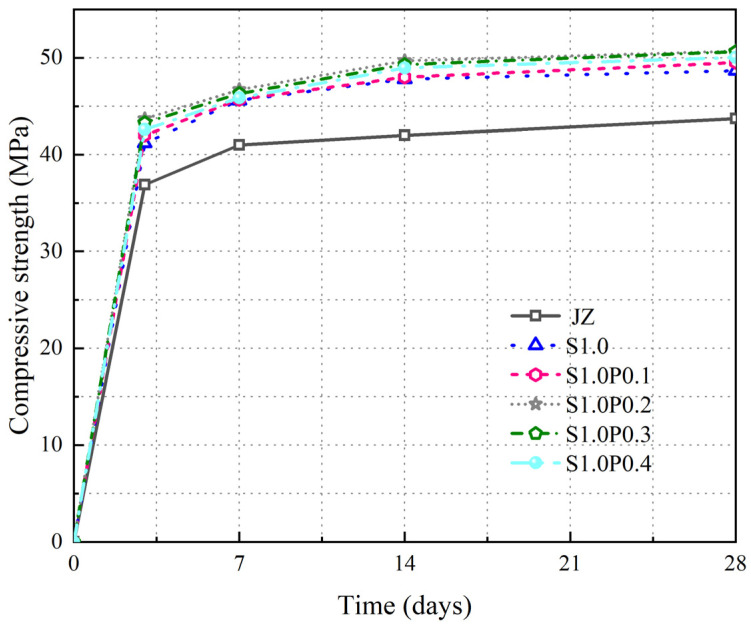
Variation of $f_{cc}$ in S-PVA HFRC.

The change in $f_{cc}$ with age for JZ, S1.0, S1.0P0.1, S1.0P0.2, S1.0P0.3, and S1.0P0.4 is shown in Figure 8.

From Figure 8, the following can be seen:

(1) With the increase in age, the $f_{cc}$ of S-PVA HFRC continued to increase.
(2) At different ages, S-PVA HF further increased the $f_{cc}$ of the single steel fiber-mixed concrete. At different ages, the $f_{cc}$ of S-PVA HFRC was highest when the steel fiber content was 1.0% and the PVA fiber content was 0.2%, which was 16% higher than the baseline concrete at 28 days. However, when the PVA fiber content was greater than 0.2%, the $f_{cc}$ of SFRC decreased.

The results show that the S-PVA HF reduces the microcracks produced in the concrete at the early stage of compression, and the PVA fibers increase the density of the concrete matrix, enhancing the pull-out resistance of steel fibers when limiting macrocracks. The hybrid effect of steel fibers and PVA fibers further improves the $f_{cc}$ of the concrete. However, when the PVA fiber content exceeds 0.2%, a large amount of agglomeration of steel fibers and PVA fibers reduces the $f_{cc}$ of the concrete. When 1.0% of steel fibers is mixed with 0.2% of PVA fibers, the $f_{cc}$ of the concrete is the highest.

From Figure 8, the observations are as follows:

(1) The $f_{cc}$ of S-PVA HFRC consistently increased with age.
(2) S-PVA HF enhanced the $f_{cc}$ of single steel fiber-mixed concrete at various ages. The peak $f_{cc}$ for S-PVA HFRC occurred at 1.0% steel fiber content and 0.2% PVA fiber content, surpassing baseline concrete by 16% at 28 days. However, increasing PVA fiber content beyond 0.2% led to a decreased concrete $f_{cc}$.

The findings suggest that S-PVA HF diminish microcracks in early-stage compressed concrete, while PVA fibers augment the concrete matrix density, thereby strengthening steel fiber pull-out resistance against macrocracks. The combined influence of steel and PVA fibers further elevates the concrete $f_{cc}$. Nonetheless, exceeding 0.2% PVA fiber content triggers steel and PVA fiber aggregation, resulting in a reduced concrete $f_{cc}$. Optimal results are achieved with a blend of 1.0% steel fibers and 0.2% PVA fibers.

### 3.2. $f_{ts}$ of Concrete

**Figure 9:**
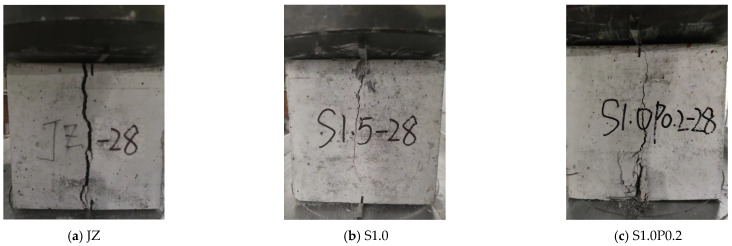
Typical test pictures of concrete $f_{ts}$ at 28 days.

The typical test figure after the failure of the concrete splitting tension is shown in Figure 9. Figure 9 displays the failure forms of the reference concrete, concrete with 1.0% steel fiber content, and concrete with 1.0% steel fiber content and a 0.2% PVA fiber content, respectively.

From Figure 9, we can infer the following:

(1) After the $f_{ts}$ test of the reference concrete, the crack width was the largest, and the concrete matrix was divided into two parts.
(2) After the $f_{ts}$ test of SFRC, the upper part of the concrete specimen in contact with the pad was crushed, the crack width was smaller, the crack line was relatively straight, and the degree of bending was not large.
(3) After the $f_{ts}$ test of the S-PVA HFRC, the lower part of the concrete specimen in contact with the pad was crushed. Meanwhile, the cracks below developed from bottom to top, the crack width was smaller, and the crack line was more fluctuating.

#### 3.2.1. Variation of $f_{ts}$ in SFRC

**Figure 10:**
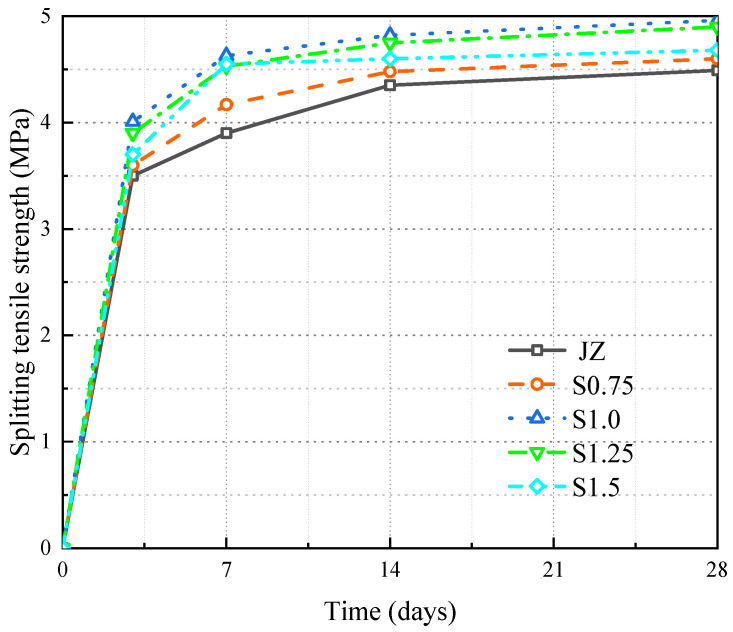
Variation of $f_{ts}$ in SFRC.

Figure 10 shows the variation in $f_{ts}$ over time for JZ, S0.75, S1.0, S1.25, and S1.5.

From Figure 10, we can observe the following:

(1) With an increasing age, there was a gradual rise in the $f_{ts}$ of the concrete. Notably, the most rapid increase occurred between days 3 and 7, reaching over 80% of the concrete’s $f_{ts}$ at day 28.
(2) At each age, $f_{ts}$ initially increased followed by a decrease as the steel fiber content grew. The peak was reached at 1.0% steel fiber content. At day 28, in comparison to JZ, the $f_{ts}$ of S1.0 rose from 4.49 MPa to 4.96 MPa, marking a 10.5% increase.

These findings highlight the positive impact of steel fiber addition on concrete $f_{ts}$ [12,14,15,16]. The bond-slip effect of steel fibers predominantly affects the tensile region. The bonding and anchoring of steel fibers to the concrete matrix substantially enhance concrete fts. However, when the steel fiber content surpasses 1.0%, $f_{ts}$’s growth plateaus. This is due to the emergence of steel fiber clusters, introducing weaker surfaces in the concrete that hamper the efficacy of the steel fibers. Generally, 1.0% steel fiber content yields the maximum $f_{ts}$ enhancement for panel concrete.

#### 3.2.2. Changes in the $f_{ts}$ of S-PVA HFRC

**Figure 11:**
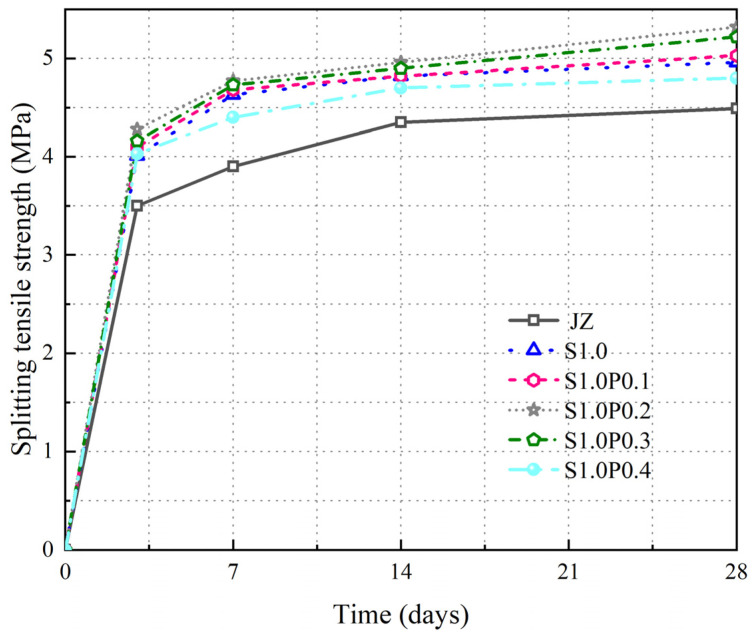
Variation of $f_{ts}$ in S-PVA HFRC.

The changes in the $f_{ts}$ over time of JZ, S1.0, S1.0P0.1, S1.0P0.2, S1.0P0.3, S1.0P0.4 are shown in Figure 11.

Figure 11 reveals the following trends:

(1) The $f_{ts}$ of S-PVA HFRC steadily increased as curing time extended.
(2) S-PVA HF additionally enhanced $f_{ts}$ for concrete featuring single steel fibers. Across different curing durations, the peak fts for S-PVA HFRC emerged at 1.0% steel fiber content and 0.2% PVA fiber content. This combination resulted in an 18.5% strength increase over the base concrete after 28 days of curing. However, surpassing 0.2% PVA fiber content led to a reduced $f_{ts}$ of concrete.

These findings underscore that the hybrid synergy and multi-layered crack mitigation capabilities of steel and PVA fibers augment SFRC $f_{ts}$. Nonetheless, excessive PVA fibers can diminish the dispersion of steel and PVA fibers within the concrete, limiting their potential and thus decreasing SFRC’s $f_{ts}$. Optimal results are achieved when concrete comprises 1.0% steel fibers and 0.2% PVA fibers, yielding the highest $f_{ts}$ for S-PVA HFRC.

### 3.3. Stress–Strain Relationship of Concrete

#### 3.3.1. Analysis of Apparent Shape in Uniaxial Compression Test of SFRC

**Figure 12:**
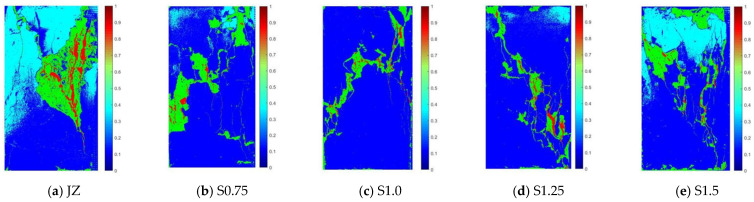
Microscopic appearance of SFRC under uniaxial compression.

The microscopic appearance of JZ concrete and S0.75, S1.0, S1.25, and S1.5 SFRC at 28 days of age (when the strain was 0.01) is shown in Figure 12.

As can be seen from Figure 12:

(1) Both the reference concrete and SFRC generated “X”-shaped through-cracks along the diagonal direction after being compressed. Many minute cracks perpendicular to the main crack appeared near the main crack.
(2) After JZ concrete was compressed, there were a lot of spalling on the surface, and the crack area was the largest. After the addition of steel fibers, the crack area was significantly reduced. The crack area and crack depth of S1.0 and S1.25 concrete were the smallest.

#### 3.3.2. Analysis of the Apparent Morphology of the Uniaxial Compression Test of S-PVA HFRC

**Figure 13:**
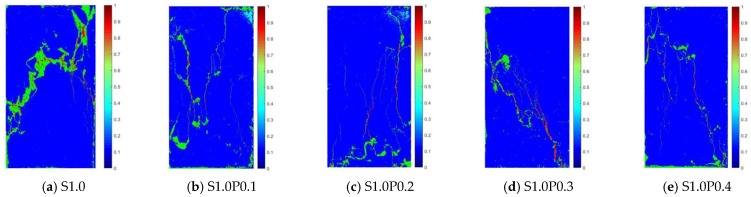
Microscopic appearance of S-PVA HFRC under uniaxial compression.

The uniaxial compression failure morphology images of S1.0, S1.0P0.1, S1.0P0.2, S1.0P0.3, S1.0P0.4 concrete at the age of 28 days (when the strain was 0.01) are shown in Figure 13.

As can be seen from Figure 13, the S-PVA HF significantly reduced the minor cracks around the main cracks, further reducing the crack area and crack depth of the concrete. The S-PVA HF notably improved the concrete’s control of cracks when the PVA fiber content was 0.1~0.4%.

#### 3.3.3. Stress–Strain Relationship of SFRC

**Figure 14:**
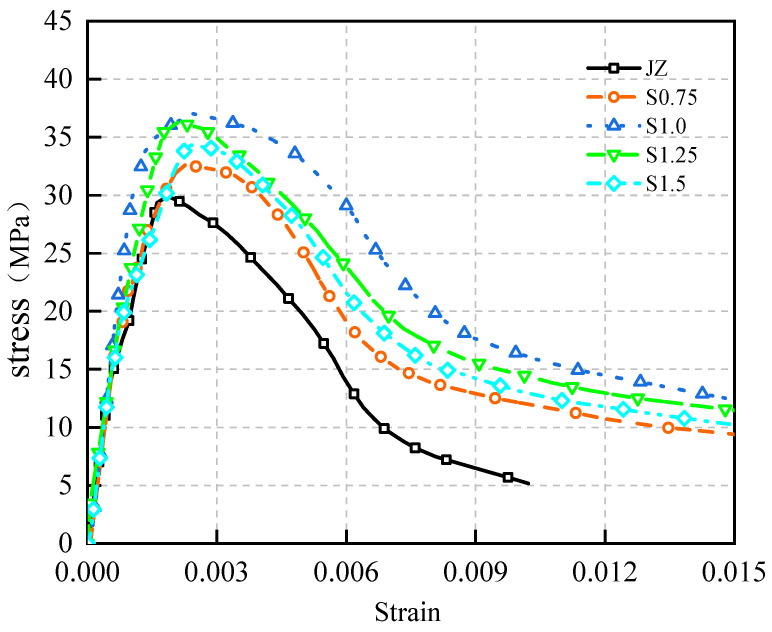
Stress–strain relationship of SFRC at 28 days.

The stress–strain curves for concrete at 28 days with steel fiber contents of 0%, 0.75%, 1.0%, 1.25%, and 1.5% are shown in Figure 14.

Figure 14 yields these observations:

(1) With increasing steel fiber content, the peak strain rose gradually, while peak stress exhibited an initial rise followed by a decrease. Notably, the introduction of steel fibers minimally affected $E_{f}$; it remained nearly identical in the curve’s initial ascent, while exerting a considerable influence on the descending phase.
(2) The area enclosed by the curve and $W_{1.0}$ initially expanded and then contracted as the steel fiber content increased, as indicated in Table 3.

These findings affirm that $E_{f}$ remains largely unaffected by steel fibers. In the presence of microcracks, steel fibers assume a bridging role post-crack formation, shouldering some stress and elevating the post-peak curve stress [42,43,44]. Nonetheless, inadequate steel fiber content impedes a robust bridging mechanism, and excessive content leads to fiber aggregation—both scenarios hinder the full potential of steel fibers’ bridging role.

**Figure 15:**
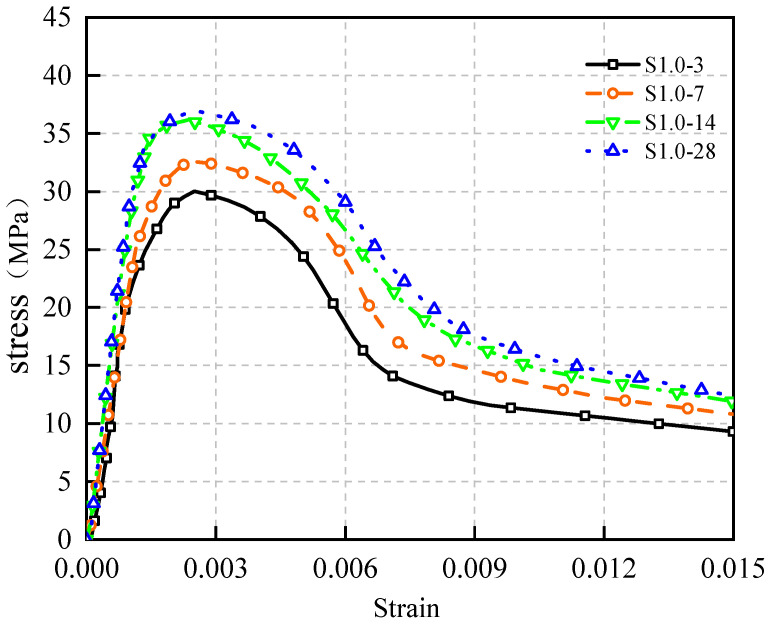
Stress–strain relationship of S1.0 from 3 days to 28 days.

The stress–strain curve of concrete with 1.0% steel fiber content from 3 days to 28 days of curing age is shown in Figure 15.

Figure 15 illustrates the following patterns:

(1) With prolonged curing age, the area encompassed by the S1.0 curve progressively enlarged, accompanied by improved $W_{1.0}$. Nevertheless, the fundamental trajectory of the curve remained largely consistent.
(2) The peak stress exhibited a gradual increment, and the incline of the curve’s rising segment experienced a slight rise. Simultaneously, the $E_{f}$ of the concrete increased, as delineated in Table 4.

These findings underscore that as cement hydration deepens over the course of curing, the concrete’s internal structure densifies, leading to heightened concrete strength, increased $E_{f}$, and elevated brittleness. Consequently, both the peak stress and the curve’s initial ascending portion witness an augmentation. Incorporating steel fibers enhances the concrete’s resilience. For the same steel fiber content, stress–strain curves at various ages exhibit minimal deviations in overall trends.

#### 3.3.4. Stress–Strain Relationship of S-PVA HFRC

**Figure 16:**
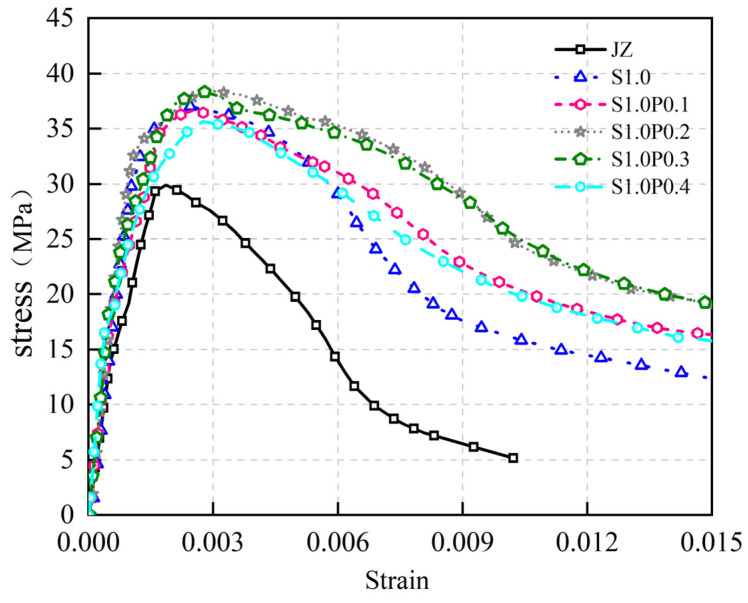
Stress–strain relationship of S-PVA HFRC at 28 days.

The stress–strain curve of JZ, S1.0, S1.0P0.1, S1.0P0.2, S1.0P0.3, and S1.0P0.4 concrete after 28 days of curing is shown in Figure 16.

Figure 16 provides the following insights:

(1) PVA fibers exerted a certain positive influence on peak stress, albeit with limitations. However, they notably augmented the slope of the curve’s rising segment. Peak stress for S1.0P0.1 and S1.0P0.4 was slightly below that of S1.0.
(2) At the 28-day mark, $W_{1.0}$ for S-PVA HFRC initially increased and then decreased with the rising PVA dosage. The largest $W_{1.0}$ for S-PVA HFRC occurred at 1.0% steel fiber content and 0.2% PVA fiber content, as detailed in Table 4.

These findings underscore that PVA fibers within S-PVA HF contribute to heightened bond strength between PVA fiber and cement. The introduction of PVA fibers densifies the matrix, marginally enhances $E_{f}$, bolsters steel fiber pull-out resistance, and augments SFRC ductility. The confluence of hybrid fiber effects and multi-level crack resistance through steel and PVA fibers further bolsters the concrete’s $W_{1.0}$.

When contrasted with previous studies, Sun [35] and Xiao [36] similarly concluded that blending steel and PVA fibers surpassed solitary mixing in enhancing the axial compression mechanics of concrete. In sum, the steel–PVA hybrid proves more effective in enhancing panel concrete ductility than standalone steel fibers.

**Figure 17:**
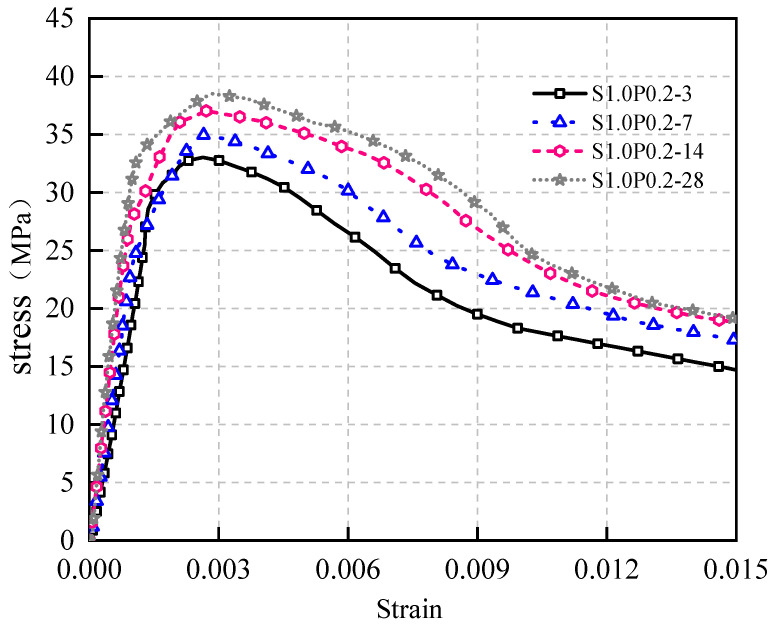
Stress–strain relationship of S1.0P0.2 from 3 days to 28 days.

The stress–strain curve for S1.0P0.2 across curing ages of 3 days to 28 days is depicted in Figure 17.

Figure 17 yields the following observations:

(1) The area enclosed by the S1.0P0.2 curve progressively expanded, concurrently improving $W_{1.0}$ as the curing age increased.
(2) A gradual escalation was discerned in peak stress and the slope of the curve’s ascending portion. $E_{f}$ likewise increased, as highlighted in Table 5. Notably, the curve’s overall trajectory closely aligned with that of S1.0.

These findings indicate that a heightened curing age corresponds with intensified cement hydration, culminating in enhanced concrete strength and $E_{f}$. Additionally, the cementitious matrix densifies, fortifying the bonding between steel and PVA fibers within the concrete matrix. This, in turn, reinforces the anchoring effect of steel fibers’ hooked ends within the matrix and augments the interaction between steel and PVA fibers. Consequently, the amplification of concrete toughness through hybrid steel–PVA fibers escalates with prolonged curing age.

### 3.4. Concrete Shrinkage Performance

#### 3.4.1. Early Shrinkage Behavior of SFRC

**Figure 18:**
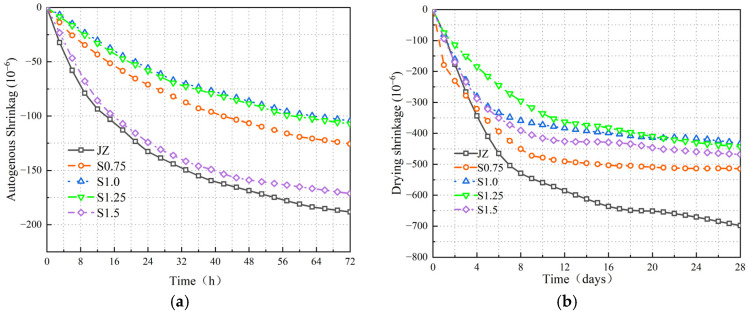
Variation of shrinkage in SFRC. (**a**) Figure of $\varepsilon_{t}$ variation at 3 days. (**b**) Figure of $\varepsilon_{d}$ variation at 28 days.

The $\varepsilon_{t}$ and $\varepsilon_{d}$ variations of JZ, S0.75, S1.0, S1.25, and S1.5 concrete are shown in Figure 18.

From Figure 18, the following can be observed:

(1) Figure 18a shows that most of the concrete’s $\varepsilon_{t}$ occurred within the first three days after initial setting. As cement hydration progressed, the $\varepsilon_{t}$ of concrete exhibited a trend of rapid decrease followed by a slower decrease.
(2) Figure 18b indicates that the $\varepsilon_{d}$ of concrete lasted for a relatively long duration, and there was still $\varepsilon_{d}$ occurring at 28 days.
(3) The inclusion of steel fibers reduced both the $\varepsilon_{t}$ and $\varepsilon_{d}$ of concrete. With an increase in the amount of steel fiber content, the $\varepsilon_{t}$ and $\varepsilon_{d}$ initially decreased and then increased. Among them, S1.0 had the smallest $\varepsilon_{d}$ rate, and at 28 days, compared to JZ, S1.0 showed a reduction of 44.5% and 38% in $\varepsilon_{t}$ and $\varepsilon_{d}$ rates, respectively.

The results demonstrate that the bond strength between steel fibers and the concrete matrix reduces the $\varepsilon_{t}$ of concrete. The addition of steel fibers and their random distribution decrease the loss of moisture and the rapid decline in internal relative humidity, thereby reducing $\varepsilon_{d}$. However, excessive steel fiber content leads to the formation of more internal weak planes, resulting in a less effective inhibition of concrete shrinkage by steel fibers.

Comparative analysis with previous research findings: Similar findings were reported by Bentley, Zheng Xiaoyan, and Shen [21,22,23,24], showing that steel fibers had a significant inhibitory effect on concrete’s $\varepsilon_{t}$ and $\varepsilon_{d}$. However, none of them identified the minimum steel fiber content for the reduction in concrete shrinkage.

#### 3.4.2. Early Shrinkage Behavior of S-PVA HFRC

**Figure 19:**
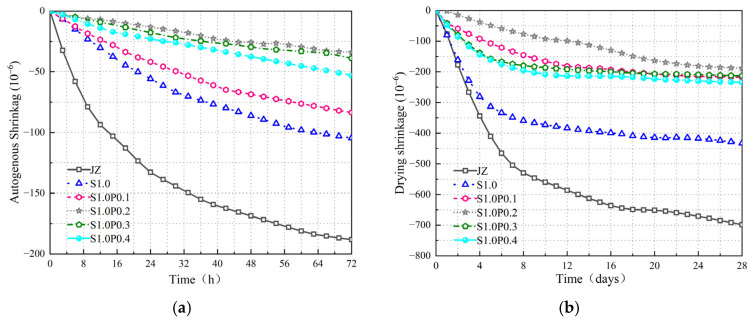
Variation of shrinkage in S-PVA HFRC. (**a**) Figure of $\varepsilon_{t}$ variation at 3 days. (**b**) Figure of $\varepsilon_{d}$ variation at 28 days.

The $\varepsilon_{t}$ and $\varepsilon_{d}$ of JZ, S1.0, S1.0P0.1, S1.0P0.2, S1.0P0.3, and S1.0P0.4 concrete are shown in Figure 19.

From Figure 19, the following can be observed:

(1) The $\varepsilon_{t}$ and $\varepsilon_{d}$ trends of S-PVA HFRC were not significantly different from those of SFRC.
(2) However, the inclusion of S-PVA HF further reduced the concrete shrinkage. As the content of PVA fibers in the hybrid mix increased, $\varepsilon_{t}$ and $\varepsilon_{d}$ initially decreased and then increased. The minimum shrinkage occurred when the concrete contained 1.0% steel fibers and 0.2% PVA fibers. Compared to S1.0, $\varepsilon_{t}$ and $\varepsilon_{d}$ were reduced by 67.6% and 56.5%, respectively.

The results indicate that PVA fibers in the steel–PVA hybrid mix exhibit better adhesion with the cementitious matrix, increasing the matrix density and enhancing the bond strength of steel fibers within the concrete. The multi-scale physical constraint provided by the S-PVA HF reduces the volume deformation of the concrete. Additionally, the formation of a water film on the surface of PVA fibers and the three-dimensional random support system formed by steel fibers within the concrete jointly reduce the flow and loss of water, thus decreasing the $\varepsilon_{d}$ of the concrete. However, excessive PVA fiber content can lead to a clustering of steel and PVA fibers, resulting in increased concrete porosity and water loss.

## 4. Comprehensive Evaluation of Concrete Cracking Resistance Performance

Based on the experimental parameter results, AHP [45,46] was used to comprehensively evaluate the concrete’s cracking resistance performance. AHP combines the relative importance sequence of the scheme layer to the target layer as the weighted values for evaluating and selecting the schemes.

Based on AHP, a cracking resistance performance judgment matrix was established for concrete. The main factors contributing to concrete shrinkage cracking include $\varepsilon_{d}$, $E_{f}$, and $f_{ts}$. $\varepsilon_{t}$ mostly occurs within the first three days after concrete casting and affects the overall shrinkage of concrete but is not the primary factor. Additionally, the $W_{1.0}$ of concrete was considered as an important indicator of concrete’s cracking resistance performance due to the compression failure experienced by panel concrete. Based on these factors, the judgment matrix in Table 5 was constructed.

Weighted results:

(2) W=[0.2571 0.2571 0.2571 0.1429 0.0858]

The preliminary test results were processed in a non-dimensional manner, considering that concrete with better cracking resistance performance had higher $f_{ts}$ and $W_{1.0}$, and lower $E_{f}$, $\varepsilon_{t}$, and $\varepsilon_{d}$. Therefore, $f_{ts}$ and $W_{1.0}$ were considered positive indicators, while $E_{f}$, $\varepsilon_{t}$, and $\varepsilon_{d}$ were considered negative indicators. Then, the range standardization transformation was applied, calculated using Formulas (3) and (4):

(3) $$A_{i}=\frac{X_{i}-\min(X_{i})}{\max(X_{i})-\min(X_{i})}(X_{i}\;\mathrm{as}\;\mathrm{positive}\;\mathrm{indicators})$$

(4) $$A_{i}=\frac{\max(X_{i})-X_{i}}{\max(X_{i})-\min(X_{i})}(X_{i}\;\mathrm{as}\;\mathrm{negative}\;\mathrm{indicators})$$

In the formula: $X_{i}$—actual value of the factor; $A_{i}$—non-dimensional value of the factor.

**Figure 20:**
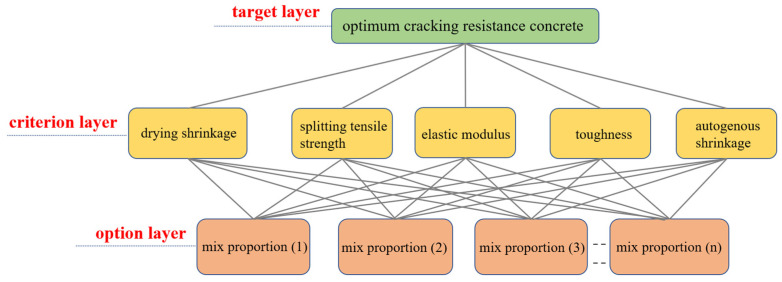
Hierarchical structure model for evaluating concrete crack resistance performance.

Based on the AHP, a multi-factor cracking resistance performance evaluation model was established. After non-dimensionalizing each factor, the comprehensive cracking resistance performance index was calculated using the weighted-sum method, as shown in Formula (5). The concrete’s cracking resistance performance was then assessed based on the magnitude of that index, as illustrated in Figure 20. Additionally, the cracking factor was used for validation, as shown in Formula (6).

(5) $$M=\sum W_{i}\times A_{i}$$

In the formula: $W_{i}$—weight of the index; $A_{i}$—non-dimensional value of the factor; M—cracking resistance index (i = 1, 2, 3, 4, 5).

(6) $$K=\frac{\delta (\varepsilon )}{f_{ts}}=\varphi \frac{E_{f}\varepsilon }{f_{ts}}$$

(7) $$M^{\prime }=\frac{1}{K}$$

In Formula (6): K—cracking factor (a larger cracking factor indicates lower cracking resistance performance); $E_{f}$—concrete elastic modulus; ε—concrete shrinkage strain value; φ—stress amplification factor due to a non-uniform restraint (assumed to be one); $f_{ts}$—concrete tensile strength (calculated using $f_{ts}$ in this paper).

In Formula (7): $M^{\prime }$—reciprocal of the cracking factor, denoted as the cracking resistance index used to evaluate the concrete’s cracking resistance performance.

### 4.1. Variation Law of Cracking Resistance Performance in SFRC

**Figure 21:**
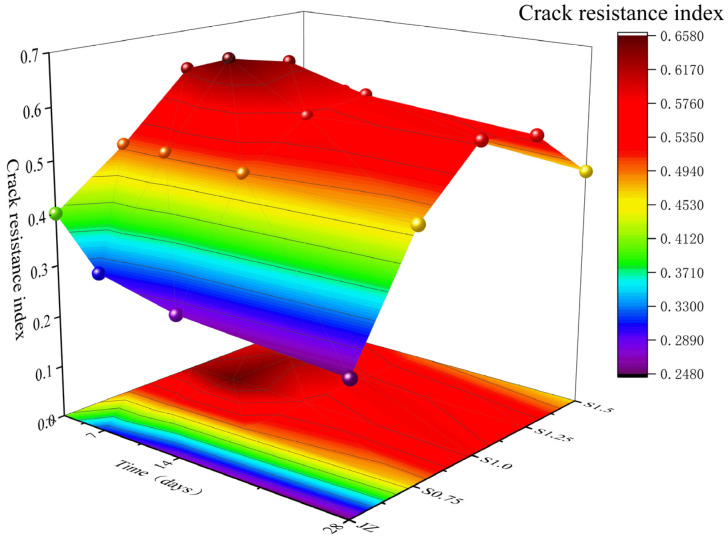
Variation in crack resistance performance in SFRC.

The cracking resistance performance indices from 3 days to 28 days for JZ, S0.75, S1.0, S1.25, and S1.5 concrete are shown in Figure 21.

From Figure 21, the following observations can be made:

(1) At different curing ages, the cracking resistance performance index of concrete generally decreased as the curing age increased.
(2) At the same curing age, the cracking resistance performance index first increased and then decreased with an increase in the steel fiber content. At 1.0% steel fiber content, the cracking resistance performance index of the SFRC was the highest. Compared to JZ, the cracking resistance performance at 3 days, 7 days, 14 days, and 28 days improved by 52.5%, 96.9%, 64.7%, and 90%, respectively.

The results indicate that as the curing age increases, the cracking resistance performance of concrete gradually decreases. This is attributed to the increasing shrinkage of concrete, the higher $E_{f}$, and an increased brittleness, leading to a decline in the concrete’s cracking resistance performance. The inclusion of steel fibers bridges macrocracks, enhancing the $f_{ts}$ and $W_{1.0}$ of concrete while reducing shrinkage. Therefore, the cracking resistance performance of the reference concrete is improved. However, excessive steel fiber content can lead to a clustering of steel fibers, causing some fibers to lose their ability to restrict crack development and reduce the concrete’s capacity to retain moisture, resulting in a decrease in cracking resistance performance.

### 4.2. Variation Law of Cracking Resistance Performance in S-PVA HFRC

**Figure 22:**
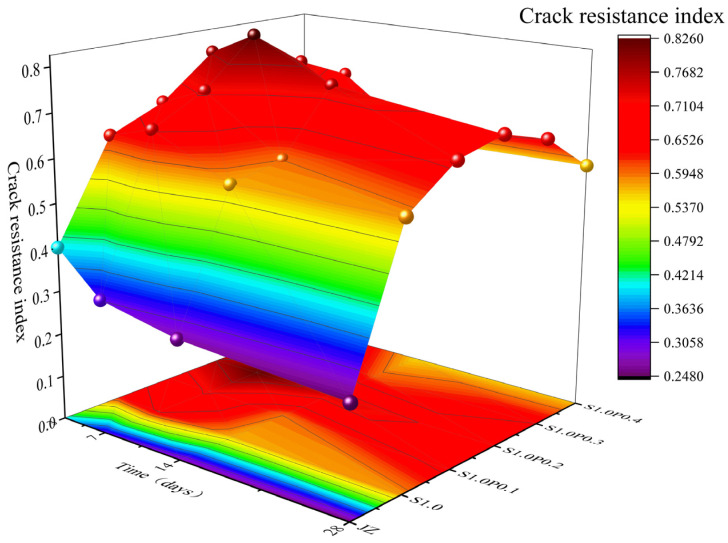
Variation of crack resistance performance in S-PVA HF.

The cracking resistance performance indices from 3 days to 28 days for JZ, S1.0, S1.0P0.1, S1.0P0.2, S1.0P0.3, and S1.0P0.4 concrete are shown in Figure 22.

From Figure 22, the following observations can be made:

(1) With the increase in curing age, the cracking resistance performance index of S-PVA HFRC gradually decreased.
(2) At the same curing age, as the PVA fiber content increased in the steel–PVA hybrid mix, the cracking resistance performance first increased and then decreased. At 0.2% PVA fiber content, S-PVA HFRC exhibited the best cracking resistance performance. Compared to S1.0, the cracking resistance performance at 3 days, 7 days, 14 days, and 28 days improved by 24.5%, 26.1%, 30%, and 19.3%, respectively. Compared to the reference concrete, the maximum improvement reached 143%.

The results indicate that the cracking resistance performance of S-PVA HFRC concrete generally decreases with the increase in curing age, consistent with the conclusions drawn from SFRC. The inclusion of S-PVA HF further enhances the concrete’s cracking resistance performance. This is because the multi-level crack-arresting ability of S-PVA HF increases the $f_{ts}$ and $W_{1.0}$ of the concrete, while the multi-scale physical constraint of S-PVA HF further reduces the shrinkage rate of the concrete, thereby improving the cracking resistance performance. However, an excessive content of PVA fibers leads to a clustering of steel and PVA fibers, resulting in a decrease in the cracking resistance performance of S-PVA HFRC.

### 4.3. $M^{\prime }$ Cracking Resistance Performance Evaluation

**Figure 23:**
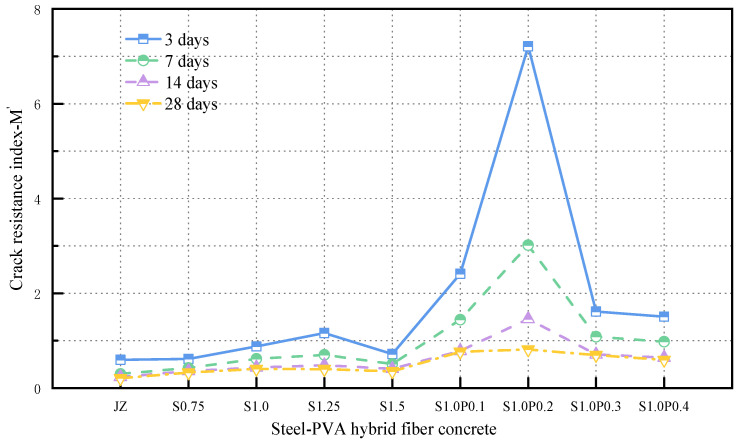
Variation in crack resistance index *M*′ in concrete.

Figure 23 shows the relationship between the concrete’s cracking resistance index calculated based on the cracking factor and the steel fiber content and PVA fiber content.

From Figure 23, the following conclusions can be drawn:

(1) With the increase in curing age, the cracking resistance index $M^{\prime }$ gradually decreased.
(2) The concrete’s cracking resistance performance was optimal when it contained 0.2% PVA fiber and 1.0% steel fiber in the hybrid mix.

The results indicate that the evaluation of concrete cracking resistance performance using the cracking factor aligns closely with the results obtained from the AHP comprehensive evaluation of cracking resistance performance. However, there might be some differences in the considered factors, and the magnitudes of the increase or decrease in the cracking resistance index may vary. Overall, the validation of the cracking resistance index calculated using the cracking factor confirms the reliability of the AHP evaluation for concrete cracking resistance performance.

## 5. Conclusions

To improve the cracking resistance of panel concrete and avoid concrete extrusion failure and shrinkage cracking, this study conducted $f_{cc}$ tests, $f_{ts}$ tests, uniaxial compression tests, drying shrinkage tests, and autogenous shrinkage tests. Based on the AHP, a multi-factor cracking resistance index evaluation model was established to comprehensively assess the cracking resistance performance of S-PVA HFRC. The main conclusions are as follows:

The inclusion of S-PVA HF further improves the $f_{cc}$, $f_{ts}$, $W_{1.0}$, and shrinkage inhibition performance of concrete. The maximum improvement in these properties is observed at 1.0% steel fiber content and 0.2% PVA fiber content in the mix.

1. Beyond three days of curing, the cracking resistance performance of concrete decreases with an increase in curing age.
2. The concrete exhibits the highest cracking resistance performance at 1.0% steel fiber content and 0.2% PVA fiber content in the mix. Compared to the reference concrete, the maximum improvement is 143%. This combination is recommended for enhancing the cracking resistance of panel concrete.
3. The evaluation model of the multi-factor cracking resistance index based on AHP is reliable, as confirmed by the validation of the cracking resistance index calculated using the cracking factor.
4. Considering the complexity of the actual working conditions of panel concrete in real water conservancy projects, more experimental studies close to the actual situation on the cracking resistance of concrete under constraint conditions are needed. This will also be the direction of future research.

## Figures and Tables

**Table 1:** Basic properties of PVA fibers.

Name	Density [g/cm^3^]	Diameter [mm]	Length [mm]	Elastic Modulus [GPa]	Tensile Strength [MPa]	Elongation [%]
RECS-15-12	1.3	0.031	12	41	1650	6

**Table 2:** Material consumption of 1 m^3^ panel concrete [unit: kg].

Test Piece Number	Water/Binder Ratio	Sand Rate	The Quantity of Materials Used for 1 m^3^ of Concrete. [Unit: kg/m^3^]								
			Water	Cement	Fly Ash	Sand	Small Stones	Large Stones	Steel Fiber	PVA Fiber	Water Reducing Agent
JZ	0.4	0.35	160	320	80	644	657.8	538.2	0	0	0.40%
S0.75	0.4	0.35	160	320	80	623.42	636.78	521	58.8	0	0.40%
S1.0	0.4	0.35	160	320	80	616.53	629.74	515.24	78.5	0	0.40%
S1.25	0.4	0.35	160	320	80	609.67	622.73	509.51	98.1	0	0.40%
S1.5	0.4	0.35	160	320	80	602.79	615.71	503.76	117.75	0	0.40%
S1.0P0.1	0.4	0.35	160	320	80	616.07	629.28	514.86	78.5	1.3	0.40%
S1.0P0.2	0.4	0.35	160	320	80	615.62	628.81	514.48	78.5	2.6	0.40%
S1.0P0.3	0.4	0.35	160	320	80	615.16	628.35	514.1	78.5	3.9	0.40%
S1.0P0.4	0.4	0.35	160	320	80	614.71	627.88	513.72	78.5	5.2	0.40%

(Note: JZ represents the base concrete, S represents steel fibers, P represents PVA fibers, and SmPn represents concrete with a $V_{f}$ of m% for steel fibers and n% for PVA fibers).

**Table 3:** Calculation table for concrete $W_{1.0}$.

$W_{1.0}$ [kN∙mm]	JZ	S0.75	S1.0	S1.25	S1.5	S1.0P0.1	S1.0P0.2	S1.0P0.3	S1.0P0.4
3 days	350.3	550.6	658.4	627.3	580.4	702.1	832.7	813.2	753.2
7 days	422.3	630.3	763.1	743.2	693.2	803.6	921.0	912.6	852.3
14 days	539.8	691.2	865.8	791.6	734.0	928.3	1038.8	1022.6	899.2
28 days	554.9	710.5	911.3	813.8	754.5	965.5	1095.5	1072.3	935.2

**Table 4:** Calculation table for concrete $E_{f}$.

$E_{f}$ [GPa]	JZ	S0.75	S1.0	S1.25	S1.5	S1.0P0.1	S1.0P0.2	S1.0P0.3	S1.0P0.4
3 days	21.3	21.0	20.0	22.3	22.0	22.3	22.3	23.0	22.8
7 days	23.9	23.1	21.5	23.8	24.0	24.2	23.1	25.0	24.1
14 days	26.1	25.1	28.2	26.3	26.3	32.8	30.2	35.2	34.2
28 days	27.5	27.3	28.3	27.6	28.3	30.1	34.7	35.1	34.5

**Table 5:** Judgment matrix for concrete crack resistance performance.

Indicator	$\varepsilon_{d}$	$f_{ts}$	$E_{f}$	$W_{1.0}$	$\varepsilon_{t}$
$\varepsilon_{d}$	1	1	1	9/5	9/3
$f_{ts}$	1	1	1	9/5	9/3
$E_{f}$	1	1	1	9/5	9/3
$W_{1.0}$	5/9	5/9	5/9	1	5/3
$\varepsilon_{t}$	3/9	3/9	3/9	3/5	1

## Data Availability

The raw data supporting the conclusions of this article will be made available by the authors on request.

## Abbreviations

AHP	Analytic Hierarchy Process
S-PVA HF	Steel–PVA hybrid fibers
SFRC	Steel fiber-reinforced concrete
HFRC	Hybrid fiber-reinforced concrete
CFRD	Concrete-faced rockfill dam
$f_{cc}$	Compressive strength
$f_{c}$	Axial compressive strength
$f_{ts}$	Splitting tensile strength
$E_{f}$	Elastic modulus of concrete
$W_{1.0}$	Area under the full axial load displacement curve with axial deformation from 0 to *L*_0_ × 1.0%mm (compression energy).
$\varepsilon_{t}$	Autogenous shrinkage
$\varepsilon_{d}$	Drying shrinkage

## References

[1] Wang Z.J., Liu S.H., Vallejo L., Wang L.J. (2014). Numerical analysis of the causes of face slab cracks in Gongboxia rockfill dam. Eng. Geol..

[2] de Pinto N.L.S. (2008). Very high CFRDs: Behaviour and design features. Int. J. Hydropower Dams.

[3] Zhou W., Hua J., Chang X., Zhou C. (2011). Settlement analysis of the Shuibuya concrete-face rockfill dam. Comput. Geotech..

[4] Qu Y.Q., Zou D.G., Kong X.J., Liu J.M., Zhang Y., Yu X. (2019). Seismic damage performance of the steel fiber reinforced face slab in the concrete-faced rockfill dam. Soil Dyn. Earthq. Eng..

[5] Baak S.H., Cho G.C., Song K.I. (2017). Stability analysis on the concrete slab of the highest concrete-faced rock-fill dam in South Korea. Geomech. Eng..

[6] (2022). The 14th Five Year Plan for Water Safety Assurance. China Water Resour..

[7] Yousefieh N., Joshaghani A., Hajibandeh E., Shekarchi M. (2017). Influence of fibers on drying shrinkage in restrained concrete. Constr. Build. Mater..

[8] Noushini A., Samali B., Vessalas K. (2013). Effect of polyvinyl alcohol (PVA) fibre on dynamic and material properties of fibre reinforced concrete. Constr. Build. Mater..

[9] Banthia N., Gupta R. (2006). Influence of polypropylene fiber geometry on plastic shrinkage cracking in concrete. Cem. Concr. Res..

[10] Zhang P., Wang K.X., Wang J., Guo J.J., Hu S.W., Ling Y.F. (2020). Mechanical properties and prediction of fracture parameters of geopolymer/alkali-activated mortar modified with PVA fiber and nano-SiO_2_. Ceram. Int..

[11] Zhang P., Zheng Y.X., Wang K.J., Zhang K.X. (2019). Combined influence of nano-CaCO_3_ and polyvinyl alcohol fibers on fresh and mechanical performance of concrete incorporating fly ash. Struct. Concr..

[12] Han J.H., Zhao M.M., Chen J.Y., Lan X.F. (2019). Effects of steel fiber length and coarse aggregate maximum size on mechanical properties of steel fiber reinforced concrete. Constr. Build. Mater..

[13] Dong S., Zhao Q., Zhu H. (2022). Mechanical properties and constitutive model of steel fiber-reinforced rubberized concrete. Constr. Build. Mater..

[14] Fu C.Q., Ye H.L., Wang K.J., Zhu K.Q., He C.Y. (2019). Evolution of mechanical properties of steel fiber-reinforced rubberized concrete (FR-RC). Compos. B Eng..

[15] Mo K.H., Yeoh K.H., Bashar I.I., Alengaram U.J., Jumaat M.Z. (2017). Shear behaviour and mechanical properties of steel fibre-reinforced cement-based and geopolymer oil palm shell lightweight aggregate concrete. Constr. Build. Mater..

[16] Abbass W., Khan M.I., Mourad S. (2018). Evaluation of mechanical properties of steel fiber reinforced concrete with different strengths of concrete. Constr. Build. Mater..

[17] Chylík R., Fládr J., Bílý P., Trtík T., Vráblík L. (2019). An analysis of the applicability of existing shrinkage prediction models to concretes containing steel fibres or crumb rubber. J. Build. Eng..

[18] Al-Kamyani Z., Figueiredo F.P., Hu H., Guadagnini M., Pilakoutas K. (2018). Shrinkage and flexural behaviour of free and restrained hybrid steel fibre reinforced concrete. Constr. Build. Mater..

[19] Han J.H., Liu Z.Y., Zhang C.F. (2023). Experimental study on impact resistance of steel-fiber-reinforced two-grade aggregate concrete. Constr. Build. Mater..

[20] Bandelj B., Saje D., Šušteršič J., Lopatič J.E., Saje F. (2011). Free shrinkage of high performance steel fiber reinforced concrete. J. Test. Eval..

[21] Miao B., Chern J.C., Yang C.A. (2003). Influences of fiber content on properties of self-compacting steel fiber reinforced concrete. J. Chin. Inst. Eng..

[22] Zheng X.Y., Ji T., Easa S.M., Zhang B.B., Jiang Z.L. (2019). Tensile basic creep behavior of lightweight aggregate concrete reinforced with steel fiber. Constr. Build. Mater..

[23] Shen D., Liu C., Kang J., Yang Q., Li M., Li C., Zeng X. (2022). Early-age autogenous Shrinkage and tensile creep of hooked-end steel fiber reinforced concrete with different thermal treatment temperatures. Cem. Concr. Compos..

[24] Shen D., Kang J., Yi X., Zhou L., Shi X. (2019). Effect of double hooked-end steel fiber on early-age cracking potential of high strength concrete in restrained ring specimens. Constr. Build. Mater..

[25] Wang J.Q., Dai Q.L., Si R.Z., Guo S.C. (2018). Investigation of properties and performances of Polyvinyl Alcohol (PVA) fiber-reinforced rubber concrete. Construct. Build. Mater..

[26] Wang Z.B., Li P.F., Han Y.D., Hao R.S., Liu W.K. (2022). Dynamic compressive properties of seawater coral aggregate concrete (SCAC) reinforced with mono or hybrid fibers. Constr. Build. Mater..

[27] Junwei Z., Shijie L., Hongjian P. (2021). Experimental investigation of multiscale hybrid fibres on the mechanical properties of high-performance concrete. Constr. Build. Mater..

[28] Wang L., Zhou S.H., Shi Y., Tang S.W., Chen E. (2017). Effect of silica fume and PVA fiber on the abrasion resistance and volume stability of concrete. Compos. B Eng..

[29] Wang Q., Zhou Z.H., Zhang J., Fang Z.S., Lai M.H. (2023). Impact of polyvinyl alcohol fiber on the full life-cycle shrinkage of cementitious composite. J. Build. Eng..

[30] Vafaei D., Ma X., Hassanli R., Duan J., Zhuge Y. (2022). Microstructural behaviour and shrinkage properties of high-strength fiber-reinforced seawater sea-sand concrete. Constr. Build. Mater..

[31] Pan Z., Zhu Y., Zhang D., Chen N., Yang Y., Cai X. (2020). Effect of expansive agents on the workability, crack resistance and durability of shrinkage-compensating concrete with low contents of fibers. Constr. Build. Mater..

[32] Afroughsabet V., Teng S. (2020). Experiments on drying shrinkage and creep of high performance hybrid-fiber-reinforced concrete. Cem. Concr. Compos..

[33] Passuello A., Moriconi G., Shah S.P. (2009). Cracking behavior of concrete with shrinkage reducing admixtures and PVA fibers. Cem. Concr. Compos..

[34] Wang L., He T.S., Zhou Y.X., Tang S.W., Tan J.J., Liu Z.T., Su J.W. (2021). The influence of fiber type and length on the cracking resistance, durability and pore structure of face slab concrete. Constr. Build. Mater..

[35] Sun L., Hao Q., Zhao J., Wu D., Yang F. (2018). Stress strain behavior of hybrid steel-PVA fiber reinforced cementitious composites under uniaxial compression. Constr. Build. Mater..

[36] Xiao L., Chen P., Huang J., Peng S., Yang Z. (2022). Compressive behavior of reinforced steel-PVA hybrid fiber concrete short columns after high temperature exposure. Constr. Build. Mater..

[37] (2015). Code for Design of Concrete Face Rockfill Dams.

[38] (2015). Code for Mix Proportion Design of Hydraulic Concrete.

[39] (2015). Steel Fiber Reinforced Concrete.

[40] (2020). Hydraulic Concrete Specification Concrete Test.

[41] (2009). Standard for Test Methods of Long-Term Performance and Durability of Ordinary Concrete.

[42] Shi X., Park P., Rew Y., Huang K., Sim C. (2020). Constitutive behaviors of steel fiber reinforced concrete under uniaxial compression and tension. Constr. Build. Mater..

[43] Iqbal S., Ali I., Room S., Khan S.A., Ali A. (2019). Enhanced mechanical properties of fiber reinforced concrete using closed steel fibers. Mater. Struct..

[44] Ran J., Li T., Chen D., Shang L., Li W., Zhu Q. (2021). Mechanical properties of concrete reinforced with corrugated steel fiber under uniaxial compression and tension. Structures.

[45] Feng Z.B., Hou H.Y., Lan H.F. (2024). Understanding university students’ perceptions of classroom environment: A synergistic approach integrating grounded theory (GT) and analytic hierarchy process (AHP). J. Build. Eng..

[46] Saaty T.L. (1990). Decision Making for Leaders: The Analytic Hierarchy Process for Decisions in a Complex World.

